# Notes by a Surgeon on Active Service

**Published:** 1915-07-24

**Authors:** 


### NOTES BY A SURGEON ON ACTIVE SERVICE.

### Circumcision Under Local Anæsthesia.

Major Porter describes a method of performing this operation which is extremely useful, since it is applicable to all cases that demand operative treatment. The technique consists in injecting 20 c.c. novocaine solution under the dorsal vessels at the root, and rotating the soft parts so as to make a ring of solution which is afterwards propelled by massage to the preputial orifice. Analgesia, complete enough to permit of the whole operation being painlessly performed, is complete after ten minutes, and the initial prick of the needle is scarcely felt (*R.A.M.C. Journal*, January 1913).

### Simple Treatment of Scabies.

After billeting in infected houses or coming into contact with infected persons, this disease is not rare among details on active service. In civil practice the most usual treatment is isolation and sulphur inunction, with copious bathing. On active service such a method will never commend itself to the medical officer, yet if untreated these cases easily spread infection, and may incapacitate a whole company. The best way of dealing with the infection is to get the detail to take a warm bath where warm water is available (where it is not, he must use cold water and a liberal application of soap), and after that to paint him over with balsam of Peru. This application smarts a little, but is most efficacious, and kills the parasite in a few hours. A second application is hardly ever needed. Under this treatment the urine should be examined a few hours after the balsam has been applied.

### Venereal Disease.

"There should be a weekly examination of all the enlisted men in the command made by the medical officers, and the company commanders should be required to be in attendance. A list of all the men found infected with venereal troubles should be made out under the direction of the company commander and posted conspicuously on the bulletin board. Among those thus affected, only those who are actually incapacitated from performing their duties should be placed on sick report, the others being required to perform all duties. Canteen and all pass privileges should be withdrawn from all whose names are thus posted, and extra severe punishment meted out to those who are detected violating this rule. As soon as anyone thus listed is, in the opinion of the surgeon, free from all manifestations of venereal infection, the surgeon should notify the company commander, so as to enable the latter to take the man's name from the list. This course cannot be construed into actual punishment of the enlisted men, for the reason that taking away canteen and pass privileges is merely in the nature of removing causes that tend to retard recovery. A weekly inspection is necessary, as there is no other way by which it can be determined whether or not individuals are infected."—*General Order to American Troops during the Occupation of Cuba.*

### Dust and Smoke Conjunctivitis.

A one-per-cent. ointment of yellow oxide of mercury is the most efficacious prophylactic against the conjunctivitis that gunners and men exposed to dust often complain of. I carry a supply of Parke, Davis metallic tubes, each of which contains a drachm of this ointment, and have found them most useful.

### The Importance of Good Boots.

"Over 30,000 Germans were incapacitated for duty during the first few weeks of the Franco-German War on account of injuries to the feet, the greater number of these being due to the issue of footwear hardened by long storage in the depôts. . . . One of the best methods I know of breaking in a new pair of boots is as follows: Soak the boots in water until every part is pliable, don while wet over a thick pair of socks, and use them for a walk of several hours; they will then have acquired the exact shape of the foot at the end of the walk. The boots should then be removed, tightly packed with dry oats, and placed in a cool place in order to dry gradually. The outside should be given a coat of cod-liver oil, which should be repeated several times at intervals. The oats are shaken out, and the boots will be found comfortable, as the oats have prevented shrinking, and no last can mould the inside of the boot as well as the foot itself."—*Captain Bridges, R.A.M.C., in R.A.M.C. Journal*, 1910.

